# Undergraduate rural medical training experiences and uptake of rural practice: a retrospective cohort study in South Australia

**DOI:** 10.1186/s12909-023-04182-8

**Published:** 2023-04-05

**Authors:** Susan Williams, David Gonzalez-Chica, Katrina Morgan, Bronwyn Herde, Lawrie McArthur, Lucie Walters

**Affiliations:** 1 Adelaide Rural Clinical School, Faculty of Health and Medical Sciences, The University of Adelaide, Nairne, South Australia 5252 Australia; 2 Adelaide Rural Clinical School and Discipline of General Practice, Faculty of Health and Medical Sciences, The University of Adelaide, Adelaide, South Australia 5005 Australia; 3 Discipline of General Practice, Faculty of Health and Medical Sciences, The University of Adelaide, Adelaide, South Australia 5005 Australia; 4 Adelaide Rural Clinical School, Faculty of Health and Medical Sciences, The University of Adelaide, Mount Gambier, South Australia 5290 Australia

**Keywords:** Undergraduate medical education, Clinical clerkship, Health workforce, Rural health

## Abstract

**Background:**

Rural medical training experiences provided by Rural Clinical Schools (RCS) can encourage future practice in rural locations. However, the factors influencing students’ career choices are not well understood. This study explores the influence of undergraduate rural training experiences on graduates’ subsequent practice location.

**Methods:**

This retrospective cohort study included all medical students who completed a full academic year at the University of Adelaide RCS training program between 2013–2018. Details of student characteristics, experiences, and preferences were extracted from the Federation of Rural Australian Medical Educators (FRAME, 2013–2018) survey and linked to graduates’ recorded practice location obtained from the Australian Health Practitioner Regulation Agency (AHPRA, January 2021). The rurality of the practice location was defined based on the Modified Monash Model (MMM 3–7) or Australian Statistical Geography Standard (ASGS 2–5). Logistic regression was used to examine associations between student rural training experiences and rural practice location.

**Results:**

A total of 241 medical students (60.1% females; mean age 23.2 ± 1.8 years) completed the FRAME survey (response rate 93.2%). Of these, 91.7% felt well supported, 76.3% had a rural-based clinician mentor, 90.4% reported increased interest in a rural career, and 43.6% preferred a rural practice location after graduation. Practice locations were identified for 234 alumni, and 11.5% were working rurally in 2020 (MMM 3–7; 16.7% according to ASGS 2–5). In adjusted analysis, the odds of working rurally were 3–4 times more likely among those with a rural background or lived the longest in a rural location, 4–12 times more likely among those preferring a rural practice location after graduation, and increased with the student’s rural practice self-efficacy score (*p*-value < 0.05 in all cases). Neither the perceived support, having a rural-based mentor, or the increased interest in a rural career were associated with the practice location.

**Conclusions:**

These RCS students consistently reported positive experiences and increased interest in rural practice after their rural training. Student reported preference for a rural career and rural practice self-efficacy score were significant predictors of subsequent rural medical practice. Other RCS could use these variables as indirect indicators of the impact of RCS training on the rural health workforce.

**Supplementary Information:**

The online version contains supplementary material available at 10.1186/s12909-023-04182-8.

## Background

The Australian Rural Clinical School Program was implemented over two decades ago and forms part of a strategic pipeline of rural training to address persistent rural workforce shortages. There are 22 rural clinical schools (RCS) currently funded by the Australian Government to provide high-quality rural health training opportunities for 25% of Commonwealth supported medical students [1]. The continued support for these programs is underpinned by evidence that rural experiences during medical school can encourage future practice in rural locations [2–4].

A key study across 12 Australian medical schools reported that RCS students were more likely to be working rurally at five years post-graduation, 1.5 or 2.6 times depending on the remoteness classification, and this influence was apparent for students of rural or metropolitan background [5]. Studies of individual RCSs have also shown positive impacts on rural practice after graduation [4, 6–9]. However, whether the RCS experience reinforces students’ pre-existing rural career interests, or inspires new interest, is not well established [10]. Furthermore, little is known about the aspects of rural training initiatives that most influence students’ intentions and career choices. Along with a rural background, there is some evidence that building rural practice self-efficacy (students’ beliefs in their capabilities to practise in rural settings) may increase student intentions for a rural medical career [11]. These beliefs may be enhanced by positive experiences during RCS.

The Adelaide Rural Clinical School (ARCS) program is offered to students in their 5^th^ (penultimate) year of medical school. Students complete a 36-week rural longitudinal integrated clinical placement program based primarily in general practice, following supervisors and patients into local hospitals [12]. During 2013 to 2018 there were around 40 students per year, distributed between eight or nine home-base learning sites ranging from inner regional to very remote locations in South Australia (SA), with one town near the South Australian border in New South Wales. An evaluation of student characteristics, experiences and career intentions is undertaken annually using the national Federation of Rural Australian Medical Educators (FRAME) exit survey [13]. How these RCS student experiences are related to practice locations following graduation has not been examined to date. The objective of this study was to examine the influence of undergraduate rural medical training experiences on graduates’ subsequent location of practice, by linking data from the FRAME exit survey to recorded practice location data from the Australian Health Practitioner Regulation Agency (AHPRA) register.

## Methods

This is a retrospective cohort study of ARCS medical alumni who completed a full academic year in the rural clinical program between 2013–2018.

### FRAME Data

The FRAME exit survey is distributed to medical students completing a year-long rural placement at an Australian RCS [13, 14]. The survey collects demographic details, measures of student experiences and satisfaction, their career intentions, and preferred practice locations. Details about the survey have been published elsewhere [13, 14]. Informed consent to participate was provided at the commencement of the survey. For this study, we used FRAME data for ARCS cohorts 2013–2018 for selected survey items that were consistent across this period (Supplementary Table S1). Data with the variables of interest were provided following the FRAME protocol approved by the Flinders University Social and Behavioural Research Ethics Committee (Project number 4098).

### AHPRA data

ARCS alumni career and location outcomes were tracked using data from AHPRA, a publicly available database that provides up-to-date information on registered medical practitioners and their self-nominated main location of current practice [15]. A complete list of the medical board database, including the full name of currently registered medical practitioners, registration number, university and year of graduation, and postcode of the main place of medical practice was obtained in January 2021, representing practice locations in 2020. ARCS alumni from 2013–2018, corresponding to post-graduation years 1–6, were identified in the AHPRA register using student records at the University of Adelaide (name and year of graduation). The University student ID was then used to link AHPRA practice location information to corresponding FRAME data for these graduates. The project received approval from the Human Research Ethics Committee of The University of Adelaide (H-2020–047).

### FRAME variables

The FRAME survey questions used in this study (Supplementary Table S1) were: (i) student demographics (gender, age, rural background, ‘type of location lived longest’), (ii) items related to RCS support (individual items and using a 5-item score), (iii) items related to ARCS experiences (had a rural-based clinical mentor, increased interest in rural medical career), (iv) items related to rural practice self-efficacy (individual items and using a 6-item score) and (v) preferred practice locations following graduation. The rural background was reported as a binary (yes/no) response. ‘Type of location lived longest’ was re-classified as ‘capital city/major urban centre’ or ‘regional/rural/remote’ based on the FRAME survey descriptors. Student experiences, perceived support, and rural practice self-efficacy questions related to the ARCS program were assessed using multiple survey items based on Likert 5-point responses (1: Strongly disagree / 2: Somewhat disagree / 3: Neutral / 4: Somewhat agree / 5: Strongly agree). Ordinal variables for these Likert responses were used for analysis. These scales were also used for: My RCS medical experience has increased my interest in pursuing a career in (i) regional or rural, or (ii) remote, Australia. Student preferred geographical location to practise after graduation survey options included 1) capital or major city, 2) inner regional city or large town (population 25,000 to 100,000), 3) small town or outer regional (10,000 to 24,999), 4) small rural or remote community (< 10,000) or 5) very remote centre/area. These were re-classified as major city/urban centre or regional/rural/remote based on the FRAME survey location descriptors.

The RCS support score was based on five questions (i) I felt well supported academically by my RCS; (ii) I felt well supported financially by my RCS; (iii) I felt academically isolated during my rural placement; (iv) I felt socially isolated during my RCS placement; (v) Overall my RCS placement impacted positively on my wellbeing. These questions used the relevant 5-point Likert scale items with reverse coding for negatively worded items. The potential support score could range from 5–25 points. A similar procedure was used to generate the 6-item rural practice self-efficacy score from the questions: (i) Rural practice is too hard; (ii) I have necessary skills to practise in a rural setting; (iii) I get a sinking (anxious) feeling when I think of working in a rural setting; (iv) I have a strong positive feeling when I think of working in a rural setting; (v) People tell me I should work in a rural setting; (vi) I see people like me taking up rural clinical practice, with reverse coding for items (i) and (iii). The potential self-efficacy scores could range from 6–30 points. The rural practice self-efficacy construct has been described previously by Isaac et al. [11].

### Outcome: medical practice location

Practice location was extrapolated from AHPRA recorded practice postcode and classified into rural or metropolitan areas using the Modified Monash Model (MMM) [16] or the Australian Statistical Geography Standard (ASGS) [17] remoteness structure classifications. Rural locations were defined as MMM 3–7 (large rural towns to very remote communities) or ASGS 2–5 (outer regional to very remote). Metropolitan locations were defined as MMM 1–2 (metropolitan areas and regional centres) or ASGS 0–1 (major city or inner regional).

### Data analysis

Depending on the FRAME variable, results were described using means, standard deviations, absolute frequencies, proportions (%), median scores and interquartile ranges. Differences in FRAME variables over time and crude results for the association between these variables and the recorded practice location were analysed using chi-square (or Fisher’s exact), ANOVA (or Kruskal–Wallis) tests of heterogeneity, or K-sample equality-of-medians tests, depending on the nature of the variable. Multivariable logistic regression models were used to determine adjusted odds ratios (OR) with 95% confidence intervals (CI) for the influence of the investigated FRAME variables on the rural location of medical practice (MMM 3–7 or ASGS 2–5). All results were adjusted for age, gender, and cohort year. Results for variables related to ARCS experiences, support, and rural self-efficacy were additionally adjusted for the location the student lived the longest. These variables were included for adjustment irrespective of the *p*-value found in crude results because of the possibility of negative or positive confounding. Determination coefficients (r2) were used to evaluate the overall model fit. All analyses were performed in STATA 17.0 (StataCorp, Texas, USA).

## Results

### Sample characteristics and ARCS experiences

Of the 257 students participating in the 5^th^ year ARCS program between 2013–2018, 241 (93.8%) completed a FRAME survey, with comparable response rates for females (92.9%) and males (92.2%). The response rates and demographic profiles for each cohort are shown in Table 1. Overall, there was a higher proportion of females in most of the years, and the median student age remained steady at 23 years. The proportion of students reporting a rural background was 31.4% overall, varying between 22.9% in 2013 and 40.5% in 2016. The proportion of students that lived the longest in a regional, rural, or remote area ranged between 12.2% in 2014 and 35.7% in 2018. Table 1 also shows that most students (76.3%) somewhat or strongly agreed in relation to having a rural-based clinical mentor; 90.4% of students somewhat or strongly agreed that the ARCS experience increased their interest in a rural medical career; and 43.6% preferred a regional, rural, or remote practice location after graduation.

**Table 1:** Comparison of ARCS alumni demographics, mentor experiences, and career preferences after completion of their fifth-year rural placement. FRAME survey data, 2013–2018

		**TOTAL n (%)**	**2013**%	**2014**%	**2015**%	**2016**%	**2017**%	**2018**%	***P*** value*
N (FRAME response rate %)		241 (93.8)	35 (97.2)	42 (100.0)	42 (93.3)	37 (80.4)	43 (97.7)	42 (95.5)	
Gender (*n* = 238)	Female	143 (60.1)	48.5	71.4	76.2	37.8	55.8	65.9	0.004
Age during rural placement in years (*n* = 239)	*Median [IQR]*	*23 *[1]	*23 *[1]	*23 *[1]	*23 *[2]	*23 *[1]	*23 *[1]	*23 *[2]	0.19**
Has a rural background (*n* = 236)	Yes	74 (31.4)	22.9	27.5	31.0	40.5	32.6	33.3	0.70
Location lived the longest (*n* = 237)	Regional/ rural/remote	63 (26.6)	20.6	12.2	27.5	35.1	27.9	35.7	0.15
Had a rural-based mentor (*n* = 240)	Agree^a^	183 (76.3)	62.9	73.8	80.5	78.4	76.7	83.3	0.38
RCS increased interest in regional/rural medical career (*n* = 239)	Agree^a^	216 (90.4)	82.4	88.1	92.7	94.6	90.7	92.9	0.59^**†**^
RCS increased interest in remote medical career (*n* = 239)	Agree^a^	115 (48.1)	47.1	33.3	53.7	46.0	65.1	42.9	0.08
Preferred location of practice after graduation (*n* = 236)	Regional/ rural/remote	103 (43.6)	44.1	23.8	52.5	44.4	58.1	39.0	0.03

*ARCS *Adelaide Rural Clinical School, *RCS *Rural clinical school, *FRAME *Federation of Rural Australian Medical Educators, *IQR *Interquartile range, *SD  S*tandard deviation

^a^Combined responses of ‘Somewhat agree’ and ‘Strongly agree’

^*^ Pearson’s Chi2 test of heterogeneity

^**^ Kruskal–Wallis test of heterogeneity

^**†**^ Fisher’s exact test

Concerning ARCS support, Table 2 shows that most students reported feeling well-supported overall (91.7%). The median support score (5-item) was high overall, with some variation between years. Most ARCS students somewhat or strongly agreed that they felt well-supported academically (89.5%) and financially (96.3%). About a third of students somewhat or strongly agreed that they felt academically or socially isolated during their placement (36.3% and 35.4%, respectively). However, 87.8% of students agreed that the RCS placement positively impacted their wellbeing.

**Table 2:** Comparison of ARCS alumni support scores and rural practice self-efficacy scores after completion of their fifth-year rural placement. FRAME survey data, 2013–2018

		**TOTAL n (%)**	**2013**%	**2014**%	**2015**%	**2016**%	**2017**%	**2018**%	***P***value*
N (FRAME response rate %)		**241 (93.8)**	**35 (97.2)**	**42 (100.0)**	**42 (93.3)**	**37 (80.4)**	**43 (97.7)**	**42 (95.5)**	
Felt well supported (overall) (*n* = 240)	Agree^c^	220 (91.7)	85.7	83.3	95.1	89.2	100.0	95.2	0.03^**†**^
*Total support score*^a^* (n* = *239)*	*Median Score [IQR]*	*20 *[6]	*18 *[6]	*19 *[5]	*20 *[5]	*21 *[4]	*22 *[4]	*20 *[5]	< 0.001**
Felt well supported (academic)^a^ (*n* = 239)	Agree^c^	214 (89.5)	82.9	76.2	97.5	91.9	95.4	92.9	0.02^**†**^
Felt well supported (financial)^a^ (*n* = 240)	Agree^c^	231 (96.3)	88.6	95.2	95.1	97.3	100.0	100.0	0.08^**†**^
Felt isolated (academic)^a^ (*n* = 240)	Agree^c^	87 (36.3)	40.0	57.1	24.4	29.7	25.6	40.5	0.02
Felt isolated (social)^a^ (*n* = 240)	Agree^c^	85 (35.4)	42.9	59.5	31.7	18.9	25.6	33.3	0.003
Positive impact on wellbeing^a^ (*n* = 238)	Agree^c^	209 (87.8)	82.9	71.4	97.5	83.3	95.4	95.2	0.001^**†**^
*Rural practice self-efficacy score*^b^* (n* = *236)*	*Mean Score* ± *SD*	*23.5* ± *3.4*	*23.0* ± *3.0*	*22.3* ± *3.4*	*24.3* ± *3.2*	*23.6* ± *3.2*	*24.3* ± *3.6*	*23.4* ± *3.3*	*0.051‡*
Rural practice is too hard^b^ (*n* = 238)	Agree^c^	15 (6.3)	17.7	4.9	0.0	5.4	7.0	4.8	0.07^**†**^
Have necessary skills^b^ (*n* = 238)	Agree^c^	175 (73.5)	70.6	63.4	75.6	81.1	79.1	71.4	0.51
Get a sinking (anxious) feeling^b^ (*n* = 237)	Agree^c^	13 (5.5)	5.9	7.3	0.0	8.1	11.6	0.0	0.07^**†**^
Strong positive feeling^b^ (*n* = 237)	Agree^c^	176 (74.3)	73.5	53.7	82.9	75.7	81.4	78.1	0.03
People tell me I should work rural^b^ (*n* = 238)	Agree^c^	165 (69.3)	73.5	58.5	63.4	73.0	79.1	69.1	0.37
See people like me in rural practice^b^ (*n* = 238)	Agree^c^	154 (64.7)	55.9	48.8	73.2	67.6	79.1	61.9	0.048

*ARCS *Adelaide Rural Clinical School, *FRAME *Federation of Rural Australian Medical Educators, *IQR *Interquartile range, *SD *Standard deviation

^a^Variables used to create the support score. Support score combines responses (1 = strongly disagree to 5 = strongly agree) for the FRAME questions 1) I felt well supported academically by my RCS, 2) I felt well supported financially by my RCS, 3) I felt academically isolated during my rural placement, 4) I felt socially isolated during my RCS placement, 5) Overall my RCS placement impacted positively on my wellbeing. Responses for questions 3 and 4 were inverted during the generation of the score

^b^Variables used to create the self-efficacy score. Self-efficacy score combines responses (1 = strongly disagree to 5 = strongly agree) for the FRAME questions 1) Rural practice is too hard, 2) I have necessary skills to practise in a rural setting, 3) I get a sinking (anxious) feeling when I think of working in a rural setting, 4) I have a strong positive feeling when I think of working in a rural setting, 5) People tell me I should work in a rural setting, 6) I see people like me taking up rural clinical practice. Responses for questions 1 and 3 were inverted to generate the score

^**c**^Combined responses of ‘Somewhat agree’ and ‘Strongly agree’

^*^Pearson’s Chi2 test of heterogeneity

^**†**^Fisher’s exact test

^**^Kruskal–Wallis test of heterogeneity

^**‡**^ANOVA test of heterogeneity

Table 2 also shows that the rural practice self-efficacy score remained steady over time. Rural practice was considered too hard by only 6.3% of students, and most agreed that they had the necessary skills (73.5%) and a strong positive feeling about working in a rural setting (74.3%). Most students also agreed with statements relating to persuasion (“people tell me I should work in a rural setting”) and vicarious learning (“I see people like me taking up rural practice”), 69.3% and 64.7%, respectively.

### ARCS experiences and subsequent rural medical practice location

Using AHPRA data from January 2021, we identified the practice locations of 234 of the 241 ARCS alumni who completed the FRAME survey between 2013–2018 (follow-up rate 97.1%). Twenty-seven (11.5%, 95% CI 8.0–16.3) of the matched ARCS alumni were working rurally according to the MMM 3–7 classification, or 39 alumni (16.7%, 95% CI 12.4–22.0) according to ASGS 2–5. Table S2 shows the number of ARCS alumni working rurally by year of graduation.

Table 3 presents the associations with the rural practice location according to the MMM 3–7 classification. In crude analysis, the variables associated with working in a rural location were having a rural background (*p* = 0.02), living longest in a regional/rural/remote location (*p* = 0.002), rural practice self-efficacy score (*p* < 0.001), and a preference for a regional/rural/remote location after graduation (*p* < 0.001). In adjusted analyses, working rurally was 3–4 times more likely among those with a rural background (OR 3.33; 95% CI 1.39–7.98) or who lived the longest in a rural location (OR 4.65; 95% CI 1.88–11.47). None of the variables related to ARCS experiences or support were associated with this outcome. However, each 1-point increase in the rural practice self-efficacy score was associated with a 31% increase in the odds of working in a rural location (OR 1.31; 95% CI 1.10–1.56). For alumni who had reported a preference to work rurally, 24% were working rurally in 2020 compared to only 2.3% for those who reported a preference for urban (*p* < 0.001). After adjustment, the odds of working rurally were 11.4 times higher among ARCS alumni who reported a preference for a rural location after graduation (95% CI 2.99–43.06). The variability in the outcome explained by these variables (r2) increased from 14.3% (model including age, gender, and place the student lived the longest) to 21.1% with the inclusion of the rural practice self-efficacy score and 26.3% when the preferred practice location was incorporated.

**Table 3:** Influence of ARCS experiences and career intentions (FRAME responses 2013–2018) on medical practice in a rural location (MMM 3–7) based on AHPRA data (January 2021)

	Working in a rural location (MMM 3–7)					
	Yes	No	Crude *P* value*	Adjusted odds ratio (OR)		
	n (%)	n (%)		OR	95% CI	*P* value*†*
AHPRA location matched ARCS alumni	27 (11.5)	207 (88.5)				
Gender (*n* = 234)						
Male	10 (10.8)	83 (89.3)		Ref	-	
Female	17 (12.1)	124 (87.9)	0.76	1.23	0.51–3.00	0.65^a^
Age during rural placement (*n* = 233)						
20–24 years	21 (10.2)	185 (89.8)		Ref	-	
25 + years	6 (22.2)	21 (77.8)	0.07	2.86	0.97–8.40	0.06^a^
Has a rural background (*n* = 229)						
No	13 (8.3)	143 (91.7)		Ref	-	
Yes	14 (19.2)	59 (80.8)	0.02	3.33	1.39–7.98	0.007^a^
Location lived the longest (*n* = 230)						
Major city/urban centre	13 (7.7)	155 (92.3)		Ref	-	
Regional/rural/remote	14 (22.6)	48 (77.4)	0.002	4.65	1.88–11.47	0.001^a^
*RCS support score (5-items)*^*c*^ * (n* = *220) Median[IQR]*	*19 *[5]	*20 *[5]	*0.70*^¶^	*0.92*	*0.80–1.06*	*0.27*^*b*^
Felt well supported (overall) (*n* = 220)	4.5 [1]^§^	5.0 [1]^§^	0.45^¶^	0.77	0.40–1.48	0.43^b^
Had a rural-based mentor (*n* = 220)	5.0 [1]^§^	4.0 [1]^§^	0.14^¶^	1.41	0.85–2.34	0.18^b^
RCS increased interest in regional/rural medical career (*n* = 220)	4.5 [1]^§^	4.0 [1]^§^	0.24^¶^	1.64	0.84–3.21	0.15^b^
RCS increased interest in remote medical career (*n* = 220)	4.0 [2]^§^	3.0 [1]^§^	0.25^¶^	1.49	0.94–2.36	0.09^b^
*Rural self-efficacy score*^*d*^* (n* = *230) Mean* ± *SD*	*25.7* ± *2.9*	*23.3* ± *3.2*	< *0.001††*	*1.31*	*1.10–1.56*	*0.003*^*b*^
Preferred location of practice after graduation (*n* = 229)						
Major city/urban centre	3 (2.3)	126 (97.7)		Ref	-	
Regional/rural/remote	24 (24.0)	76 (76.0)	< 0.001	11.35	2.99–43.06	< 0.001^b^

ARCS Adelaide Rural Clinical School, RCS Rural clinical school, FRAME Federation of Rural Australian Medical Educators, AHPRA Australian Health Practitioner Regulation Agency, MMM Modified Monash Model, IQR Interquartile range, SD standard deviation

^¶^K-sample equality-of-medians test with cases at the median split evenly between the above and below groups

^a^Model adjusted for gender, age group, and ARCS cohort year

^b^Model adjusted for gender, age group, ARCS cohort year and the location they lived the longest

^c^Support score combines responses (1 = strongly disagree to 5 = strongly agree) for the FRAME questions 1) I felt well supported academically by my RCS, 2) I felt well supported financially by my RCS, 3) I felt academically isolated during my rural placement, 4) I felt socially isolated during my RCS placement, 5) Overall my RCS placement impacted positively on my wellbeing. Responses for questions 3 and 4 were inverted during the generation of the score

^d^Self efficacy score combines responses (1 = strongly disagree to 5 = strongly agree) for the FRAME questions 1) Rural practice is too hard, 2) I have necessary skills to practise in a rural setting, 3) I get a sinking (anxious) feeling when I think of working in a rural setting, 4) I have a strong positive feeling when I think of working in a rural setting, 5) People tell me I should work in a rural setting, 6) I see people like me taking up rural clinical practice. Responses for questions 1 and 3 were inverted during the generation of the score

^*^ Pearson’s Chi2 test of heterogeneity; *†* Likelihood-ratio test

^§^ Median Likert scale response [interquartile range] from 1: Strongly disagree; 2: Somewhat disagree; 3: Neutral; 4: Somewhat agree; 5: Strongly agree

^*††*^ T-test

Similar results were observed when the rurality of the practice location was based on the ASGS 2–5 classification (Table S3). In this case, the r2 increased from 11.0% to 14.0% with the inclusion of the rural practice self-efficacy score and 16.8% after the preferred practice location was included.

## Discussion

According to the available literature, this is the first cohort study to examine the associations between RCS student experiences and practice location after graduation by linking FRAME survey data with AHPRA records. The results indicate that the student rural practice self-efficacy score and a reported preference for a rural career are important predictors of subsequent rural practice among RCS alumni. This evidence is informative for student rural training initiatives and has implications for the broader pipeline of rural medical workforce training.

The concept of rural practice self-efficacy has previously been described in relation to medical students [11, 18, 19] and among medical graduates [20]. Studies using national FRAME data have demonstrated positive associations between students’ rural practice self-efficacy and reported preference to work rurally [11] or in remote practice [21] which is consistent with our findings. A similar self-efficacy construct was used in a survey including 102 out of 383 medical graduates who completed a RCS training program at another South Australian university between 1997–2016 [20]. In that study, higher levels of rural practice self-efficacy were found among doctors who were working in smaller towns and communities. Moreover, rural practice self-efficacy was also directly associated with an intention to remain or return to small rural practice [20]. Our results provide further evidence that students’ rural practice self-efficacy is an important indicator of rural predisposition and, importantly, of subsequent rural practice. It is encouraging that, at the completion of their training, almost three quarters of ARCS students reported sources of self-efficacy—feeling positive about rural practice and feeling they had the necessary skills. Other sources of self-efficacy, verbal persuasion and vicarious learning, were also reported by most students. Although there is no counterfactual, it is likely that RCS experiences foster students’ belief in their capacity for rural practice. Therefore, future program improvement could be informed by a deeper understanding of what aspects of RCS placements have the greatest impact on rural practice self-efficacy. ARCS students’ self-efficacy scores did not alter meaningfully over the six-year study period suggesting there was no influence from any alterations to the course structure during this time.

Also of interest, is how rural practice self-efficacy may be related to the phenomenon of clinical courage that has been described in relation to rural doctors from around the world [22, 23]. Clinical courage has been described as the way rural doctors experience their work; how they manage the tension between practising comfortably within their familiar scope of practice and stretching themselves to meet the needs of their patients in the context where access to specialised care and resources can be limited.

Another important finding from this study is that students’ reported preference for rural practice was the strongest indicator of future rural practice, independently of the place they lived the longest (rural or urban). A stated rural intent has been a common measure when evaluating outcomes of rural training programs but has not previously been shown to reflect subsequent practice location. The preference to work rurally remained relatively steady at around 40–50% in this cohort. We found that, of those students who reported a rural preference, 24% were working rurally in 2020 (MMM classification; 30% according to ASGS), compared to only 2% among those who preferred a metropolitan area (7% for ASGS). This is a positive finding for graduates at an early career stage (PGY1-6), when considering the usual time taken for graduates to return to rural locations [24, 25]. The finding confirms student reported rural preference is a useful indicator for RCS program evaluation, and highlights the importance of providing rural internships and fellowship training opportunities to capitalise on rural intentions formed in medical school. These results are also consistent with previous studies that examined students’ rural intent on entry to medical school and subsequent rural practice. Using data from the Medical Schools Outcome Database, Herd et al. [26] found that rural intent at medical school commencement strongly determined graduates' rural preferences and rural rotations in PGY1 and PGY3. Similarly, a study of University of Western Australia graduates concluded that rural intent on entry interacts with RCS exposure to predict subsequent rural practice in PGY2-5 [27].

There were several aspects of the RCS experience that were not associated with subsequent rural practice: feeling well-supported, having a rural-based mentor, and reporting an increased interest in a rural career. Interestingly, there are some similarities between these findings and those reported by Raftery et al. [21] regarding student interest in remote practice. Using national FRAME data from 2013–2017, Raftery et al. found that student satisfaction and an increased interest in *remote* practice were not associated with intent to work *remotely*. Similarly, having a rural mentor was not related to remote practice intent [21]. Although having a rural mentor is regarded as an important support for students [28, 29] the strength of the mentoring relationship is also an important consideration, and this aspect may not be captured in the FRAME survey.

In addition to the follow-up of alumni, this study provides an evaluation of the ARCS program over a 6-year period, which is informative for quality improvement [18]. In the main, student satisfaction and experiences were consistently positive, however, social and academic isolation were reported by about one third of students over this time. For social isolation, the level is comparable to that reported nationally in 2012 (37.1%) [30] and in 2015 (31.3%) [19]. Notably, perceived social isolation has been found to be negatively associated with intent to work rurally [30], but the effect was reduced by students’ reported self-efficacy [19]. Exploring students’ perceptions about social isolation could inform strategies to address this issue, and whether there are potential benefits from the increasing levels of online teaching and digital connectivity.

The strengths of this study include the high student response rate in FRAME surveys (> 90% over the study period), and the high follow-up rate of graduates, with practice locations identified for 97% of FRAME respondents. However, as FRAME data were only available from 2013, the longest follow-up time was for graduates in PGY6. Many alumni are still in training in these early PGYs and are not well established in a working location [31, 32]. Furthermore, as the FRAME survey measures students’ intentions and self-efficacy at the completion of their placement, we cannot measure how RCS influenced these characteristics, and our results are interpreted accordingly. The AHPRA database, which has a reported accuracy of 90% [32], only records the primary place of practice, therefore we could not include any secondary rural practice locations. AHPRA data may also under-report the rural practice locations for doctors in postgraduate GP training programs. It is likely that a longer follow-up would provide more stable practice locations. Additionally, a larger sample size would allow for a comparison across discrete regional, rural, and remote MMM categories. This study included RCS alumni from one medical school and without a comparison group (i.e. those not involved in a RCS program). Although our findings cannot be generalised to all RCSs, they are relevant to other sites with similar models for rural training. Finally, while RCS experiences can promote students’ interest in rural practice, ultimately their specialty choice and vocational training experiences beyond RCS [25, 33], and their personal/family considerations [34], may become dominant factors that influence doctors’ practice locations.

## Conclusion

For these RCS alumni, a preference for a rural career reported as a student and the rural practice self-efficacy score were significant predictors of subsequent rural medical practice. These variables may be useful indicators of the future impact of RCS training on the rural health workforce. Rural training initiatives could benefit from a deeper understanding of how rural experiences can influence students’ rural practice intent and their rural practice self-efficacy.

## Supplementary Information

**Additional file 1:Table S1.** FRAME survey questions selected for analysis, 2013-2018.

**Additional file 2:Table S2.** Number of ARCS alumni with medical practice in a rural location (MMM 3-7 or ASGS 2-5) by year of graduation, AHPRA data (January 2021).

**Additional file 3:Table S3.** ARCS experiences and career intentions (FRAME responses 2013-2018) and medical practice in a rural location (ASGS 2-5) based on AHPRA data (January 2021).

## Data Availability

The datasets used and analysed during the current study are not publicly available but were made available with permission from the Federation of Rural Australian Medical Educators (FRAME). With permission from FRAME, these datasets are available from the corresponding author on reasonable request.

## Abbreviations

AHPRA: Australian Health Practitioner Regulation Agency
ANOVA: Analysis of variance
ARCS: Adelaide Rural Clinical School
ASGS: Australian Statistical Geography Standard
CI: Confidence interval
FRAME: Federation of Rural Australian Medical Educators
GP: General practitioner
MMM: Modified Monash Model
OR: Odds ratio
PGY: Post-graduate year
RCS: Rural clinical school
