# Supplementary material for: Undergraduate rural medical training experiences and uptake of rural practice: a retrospective cohort study in South Australia
Source: BMC Med Educ. 2023 Apr 5;23:217. doi: 10.1186/s12909-023-04182-8 (PMC10077608; doi:10.1186/s12909-023-04182-8)
Supplement: Supplementary file 1 — Additional file 1:Table S1. FRAME survey questions selected for analysis, 2013-2018. [file 12909_2023_4182_MOESM1_ESM.docx]

Table S1 – FRAME survey questions, 2013-2018

| **Questions** | **Options as presented in the FRAME survey** |
| --- | --- |
| Gender | Male / Female |
| Date of birth^†^ | dd/mm/yyyy |
| Do you consider yourself to come from a rural background? | No / Yes |
| Type of location you have lived in the longest in Australia | Capital city  Major urban centre (>100,000)  Regional city/large town (25,000-100,000)  Smaller town (10,000-24,999)  Small rural community (<10,000)  Remote centre/area |
| Overall I felt well supported by my RCS | Strongly disagree / Somewhat disagree / Neutral / Somewhat agree / Strongly agree |
| I have a rural-based clinician as a mentor | Strongly disagree / Somewhat disagree / Neutral / Somewhat agree / Strongly agree |
| My RCS medical experience has increased my interest in pursuing a career in regional or rural Australia (RA2-3) | Strongly disagree / Somewhat disagree / Neutral / Somewhat agree / Strongly agree |
| My RCS medical experience has increased my interest in pursuing a career in remote and very remote Australia (RA4-5) | Strongly disagree / Somewhat disagree / Neutral / Somewhat agree / Strongly agree |
| ***Support score questions*** |  |
| I felt well supported academically by my RCS | Strongly disagree / Somewhat disagree / Neutral / Somewhat agree / Strongly agree |
| I felt well supported financially by my RCS | Strongly disagree / Somewhat disagree / Neutral / Somewhat agree / Strongly agree |
| I felt academically isolated during my rural placement^¶^ | Strongly disagree / Somewhat disagree / Neutral / Somewhat agree / Strongly agree |
| I felt socially isolated during my RCS placement^¶^ | Strongly disagree / Somewhat disagree / Neutral / Somewhat agree / Strongly agree |
| Overall my RCS placement impacted positively on my wellbeing | Strongly disagree / Somewhat disagree / Neutral / Somewhat agree / Strongly agree |
| ***Rural self-efficacy score questions*** |  |
| Rural practice is too hard^¶^ | Strongly disagree / Somewhat disagree / Neutral / Somewhat agree / Strongly agree |
| I have necessary skills to practise in a rural setting | Strongly disagree / Somewhat disagree / Neutral / Somewhat agree / Strongly agree |
| I get a sinking (anxious) feeling when I think of working in a rural setting^¶^ | Strongly disagree / Somewhat disagree / Neutral / Somewhat agree / Strongly agree |
| I have a strong positive feeling when I think of working in a rural setting | Strongly disagree / Somewhat disagree / Neutral / Somewhat agree / Strongly agree |
| People tell me I should work in a rural setting | Strongly disagree / Somewhat disagree / Neutral / Somewhat agree / Strongly agree |
| I see people like me taking up rural clinical practice | Strongly disagree / Somewhat disagree / Neutral / Somewhat agree / Strongly agree |
| In which geographical location within Australia would you most like to practise on completing your training? | Capital city  Major urban centre (>100,000)  Regional city/large town (25,000-100,000)  Smaller town (10,000-24,999)  Small rural community (<10,000)  Remote centre/area |

^†^ Age was calculated as the difference between the reported year of birth and the year of the FRAME survey

^¶^ Reverse coding used for these variables during the generation of the corresponding scores
