# Supplementary material for: Undergraduate rural medical training experiences and uptake of rural practice: a retrospective cohort study in South Australia
Source: BMC Med Educ. 2023 Apr 5;23:217. doi: 10.1186/s12909-023-04182-8 (PMC10077608; doi:10.1186/s12909-023-04182-8)
Supplement: Supplementary file 2 — Additional file 2:Table S2. Number of ARCS alumni with medical practice in a rural location (MMM 3-7 or ASGS 2-5) by year of graduation, AHPRA data (January 2021). [file 12909_2023_4182_MOESM2_ESM.docx]

**Table S2.** Number of ARCS alumni with medical practice in a rural location (MMM 3-7 or ASGS 2-5) by year of graduation, AHPRA data (January 2021).

|  | Year graduated  (PGY in 2020) | | | | | |  |
| --- | --- | --- | --- | --- | --- | --- | --- |
|  | 2014  (PGY 6) | 2015  (PGY 5) | 2016  (PGY 4) | 2017  (PGY 3) | 2018  (PGY 2) | 2019  (PGY 1) | Total |
| ARCS alumni | 32 | 41 | 42 | 37 | 43 | 39 | 234 |
| Working MMM 3-7 (%) | 8 (25.0) | 3 (7.3) | 7 (16.7) | 3 (8.1) | 4 (9.3) | 2 (5.1) | 27 (11.5) |
| Working ASGS 2-5 (%) | 9 (28.1) | 3 (7.3) | 10 (23.8) | 6 (16.2) | 7 (16.3) | 4 (10.3) | 39 (16.7) |

Fisher’s exact tests for heterogeneity: p=0.121 (MMM 3-7); p=0.141 (ASGS 2-5)
