# Supplementary material for: Undergraduate rural medical training experiences and uptake of rural practice: a retrospective cohort study in South Australia
Source: BMC Med Educ. 2023 Apr 5;23:217. doi: 10.1186/s12909-023-04182-8 (PMC10077608; doi:10.1186/s12909-023-04182-8)
Supplement: Supplementary file 3 — Additional file 3:Table S3. ARCS experiences and career intentions (FRAME responses 2013-2018) and medical practice in a rural location (ASGS 2-5) based on AHPRA data (January 2021). [file 12909_2023_4182_MOESM3_ESM.docx]

**Table S3.** ARCS experiences and career intentions (FRAME responses 2013-2018) and medical practice in a rural location (ASGS 2-5) based on AHPRA data (January 2021).

|  | Working in a rural location (ASGS 2-5) | | | | | |
| --- | --- | --- | --- | --- | --- | --- |
|  | Yes | No | Crude  p-value* | Adjusted odds ratio (OR) | | |
|  | n (%) | n (%) |  | OR | 95% CI | P value*†* |
| AHPRA location matched ARCS alumni | 39 (16.7) | 195 (83.3) |  |  |  |  |
| Gender (n=234) |  |  |  |  |  |  |
| Male | 17 (18.3) | 76 (81.7) |  | Ref | - |  |
| Female | 22 (15.6) | 119 (84.4) | 0.59 | 0.87 | 0.41-1.82 | 0.71^a^ |
| Age during rural placement (n=233) |  |  |  |  |  |  |
| 20-24 years | 32 (15.5) | 174 (84.5) |  | Ref | - |  |
| 25+ years | 7 (25.9) | 20 (74.1) | 0.17 | 1.87 | 0.71-4.97 | 0.21^a^ |
| Has a rural background (n=229) |  |  |  |  |  |  |
| No | 18 (11.5) | 138 (88.5) |  | Ref | - |  |
| Yes | 20 (27.4) | 53 (72.6) | 0.003 | 3.54 | 1.66-7.58 | 0.001^a^ |
| Location lived the longest (n=230) |  |  |  |  |  |  |
| Major city/urban centre | 19 (11.3) | 149 (88.7) |  | Ref | - |  |
| Regional/rural/remote | 19 (30.7) | 43 (69.4) | <0.001 | 4.04 | 1.86-8.74 | <0.001^a^ |
| *RCS support score (5-items)^c^ (n=220) Median[IQR]* | *19[5]* | *20[5]* | *0.45*^¶^ | *0.91* | *0.80-1.03* | *0.12^b^* |
| Felt well supported (overall) (n=220) | 5[1]^§^ | 5[1]^§^ | 0.99^¶^ | 1.05 | 0.57-1.94 | 0.88^b^ |
| Had a rural-based mentor (n=220) | 4.5[1]^§^ | 4[1]^§^ | 0.62^¶^ | 1.02 | 0.71-1.46 | 0.93^b^ |
| RCS increased interest in regional/rural medical career (n=220) | 4[1]^§^ | 4[1]^§^ | 0.40^¶^ | 1.37 | 0.79-2.37 | 0.27^b^ |
| RCS increased interest in remote medical career (n=220) | 4[1]^§^ | 3[1]^§^ | 0.19^¶^ | 1.42 | 0.96-2.10 | 0.08^b^ |
| *Rural self-efficacy score^d^ (n=230) Mean ± SD* | *25.1 ± 3.2* | *23.3 ± 3.2* | *0.001††* | *1.17* | *1.02-1.35* | *0.02^b^* |
| Preferred location of practice after graduation (n=229) |  |  |  |  |  |  |
| Major city/urban centre | 9 (7.0) | 120 (93.0) |  | Ref | - |  |
| Regional/rural/remote | 30 (30.0) | 70 (70.0) | <0.001 | 3.99 | 1.63-9.74 | 0.002^b^ |

ARCS – Adelaide Rural Clinical School; RCS – Rural clinical school; FRAME - Federation of Rural Australian Medical Educators; AHPRA - Australian Health Practitioner Regulation Agency; ASGS - Australian Statistical Geography Standard remoteness structure; IQR – interquartile range; SD – standard deviation

* Pearson’s Chi2 test of heterogeneity; *†* Likelihood-ratio test

¶ K-sample equality-of-medians test with cases at the median split evenly between the above and below groups

§ Median Likert scale response (interquartile range) from 1: Strongly disagree; 2: Somewhat disagree; 3: Neutral; 4: Somewhat agree; 5: Strongly agree

*†† T-test*

a – Model adjusted for gender, age group, and ARCS cohort year

b – Model adjusted for gender, age group, ARCS cohort year and the location they lived the longest

c – Support score combines responses (1=strongly disagree to 5=strongly agree) for the FRAME questions 1) I felt well supported academically by my RCS, 2) I felt well supported financially by my RCS, 3) I felt academically isolated during my rural placement, 4) I felt socially isolated during my RCS placement, 5) Overall my RCS placement impacted positively on my wellbeing. Responses for questions 3 and 4 were inverted during the generation of the score.

d – Self-efficacy score combines responses (1=strongly disagree to 5=strongly agree) for the FRAME questions 1) Rural practice is too hard, 2) I have necessary skills to practise in a rural setting, 3) I get a sinking (anxious) feeling when I think of working in a rural setting, 4) I have a strong positive feeling when I think of working in a rural setting, 5) People tell me I should work in a rural setting, 6) I see people like me taking up rural clinical practice. Responses for questions 1 and 3 were inverted during the generation of the score.
